# Population genomics and climate adaptation of a C4 perennial grass, *Panicum hallii* (Poaceae)

**DOI:** 10.1186/s12864-018-5179-7

**Published:** 2018-11-01

**Authors:** Billie A. Gould, Juan Diego Palacio-Mejia, Jerry Jenkins, Sujan Mamidi, Kerrie Barry, Jeremy Schmutz, Thomas E. Juenger, David B. Lowry

**Affiliations:** 1 Myriad Women’s Health, South San Francisco, CA 94080 USA; 2 Department of Plant Biology, Michigan State University, East Lansing, MI 48824 USA; 3 Great Lakes Bioenergy Research Center, Michigan State University, East Lansing, MI 48824 USA; 4 Department of Integrative Biology, University of Texas at Austin, Austin, TX 78712 USA; 5 Genome Sequencing Center, HudsonAlpha Institute for Biotechnology, Huntsville, AL 35806 USA; 6 Department of Energy, Joint Genome Institute, Walnut Creek, CA 94598 USA; 7 Plant Resilience Institute, Michigan State University, East Lansing, MI 48824 USA

**Keywords:** Population genomics, Climate GWAS, Ecotypes, Local adaptation, Grasses

## Abstract

**Background:**

Understanding how and why genetic variation is partitioned across geographic space is of fundamental importance to understanding the nature of biological species. How geographical isolation and local adaptation contribute to the formation of ecotypically differentiated groups of plants is just beginning to be understood through population genomic studies. We used whole genome sequencing combined with association study of climate to discover the drivers of differentiation in the perennial C4 grass *Panicum hallii*.

**Results:**

Sequencing of 89 natural accessions of *P.hallii* revealed complex population structure across the species range. Major population genomic separation was found between subspecies *P.hallii var. hallii* and *var. filipes* as well as between at least four major unrecognized subgroups within *var. hallii*. At least 139 genomic SNPs were significantly associated with temperature or precipitation across the range and these SNPs were enriched for non-synonymous substitutions. SNPs associated with temperature and aridity were more often found in or near genes than expected by chance and enriched for putative involvement in dormancy processes, seed maturation, response to hyperosmosis and salinity, abscisic acid metabolism, hormone metabolism, and drought recovery.

**Conclusions:**

Both geography and climate adaptation contribute significantly to patterns of genome-wide variation in *P.hallii*. Population subgroups within *P.hallii* may represent early stages in the formation of ecotypes. Climate associated loci identified here represent promising targets for future research in this and other perennial grasses.

**Electronic supplementary material:**

The online version of this article (10.1186/s12864-018-5179-7) contains supplementary material, which is available to authorized users.

## Background

Understanding how and why genetic variation is partitioned across geographic space is of fundamental importance to understanding the nature of biological species [1–3]. For widespread species, the combination of geography and local adaptation is well known to contribute to differentiation of populations and species across space [4, 5]. In plants, the evolution of locally adapted ecotypes in distinct geographic regions is common [2, 5, 6]. Those ecogeographically partitioned ecotypes typically constitute genetically structured groups [7], which are reproductively isolated from each other to varying degrees (but see [8]). While general patterns in the partitioning of plant species across space is well established for many species, an understanding of the extent to which different ecotypes overlap in their ranges and the distribution of locally adapted alleles across those groups is just beginning to emerge [7]. Overall, studies of population genomic variation in individual plant species can provide insights into the patterns and drivers of genetic divergence across particular geographic regions that vary in climate.

Hall’s panicgrass, *Panicum hallii* Vasey, is a perennial C4 grass and a member of the Panicoid grasses, which includes sorghum, maize, and sugarcane [9]. *P. hallii* is a diploid model system for the closely related tetraploid bioenergy crop switchgrass (*Panicum virgatum*). Beyond having a favorable ploidy for genetics, *P. hallii* has a short generation time, a small stature, a moderate sized genome (~ 550 Mbp), and a primarily self-fertilizing mating system [10, 11]. Just like switchgrass, which is composed of two major ecotypes (upland and lowland [12]), *P. hallii* has two major ecotypic subspecies (varieties), *P. hallii var. hallii* (hereafter *var. hallii*) and *P.hallii var. filipes* (hereafter *var. filipes*) [13, 14]. Similar to upland switchgrass, *var. hallii* is typically found in drier habitats across the range of the species, which stretches from Arizona to central Texas and south into northern Mexico. *P. hallii var. filipes*, like lowland switchgrass, is typically found in more mesic habitats and is restricted to the southern Rio Grande Valley and the Gulf-Coast of South Texas and Northern Mexico. In general, *var. hallii* is smaller in stature, has fewer tillers, has larger seeds, and flowers earlier than *var. filipes* [12, 15]. The two varieties are cross compatible and quantitative trait locus (QTL) analyses have revealed that large-effect, potentially pleiotropic, QTLs are responsible for morphological divergence between them [15]. Further, the two varieties have distinct patterns of physiological and gene expression responses to soil water availability under field conditions [16].

A recent microsatellite-based population structure analysis by Lowry et al. [11] suggested that *var. hallii* is further subdivided into two subgroups, one occurring primarily in low elevation regions of Texas, with the other occupying higher elevation sky islands of Chihuahuan and Sonoran deserts. Thus, the population structure of *P. hallii* is likely more complex than the major division between the two recognized varieties. Despite this new insight, the previous study by Lowry et al. [11] utilized only 15 genetic markers and therefore could not distinguish fine-scale structure or identify loci that might have experienced natural selection across the range of *P. hallii*. Further, the previous study only had one accession from northern Mexico, which represents a large portion of *P. hallii’s* range, and only three accessions of *var. filipes*.

In this study, we conducted whole genome resequencing of accessions of *P. hallii* from 89 locations to better understand genomic variation and patterns of population structure and across its range. With whole genome resequencing, we were afforded the opportunity to identify potential candidate genes and genomic regions that could be involved in local adaptation to different ecoregions. Overall, our goals were: 1) To characterize fine-scale population structure and hybridization between and within *P.hallii* subgroups; 2) To identify regions of the genome associated with local adaptation to major climate variation in temperature and precipitation; and 3) To provide a genomic resource for future studies into the population genomics of perennial grasses.

## Results

### Population divergence and structure

**Fig. 1:**
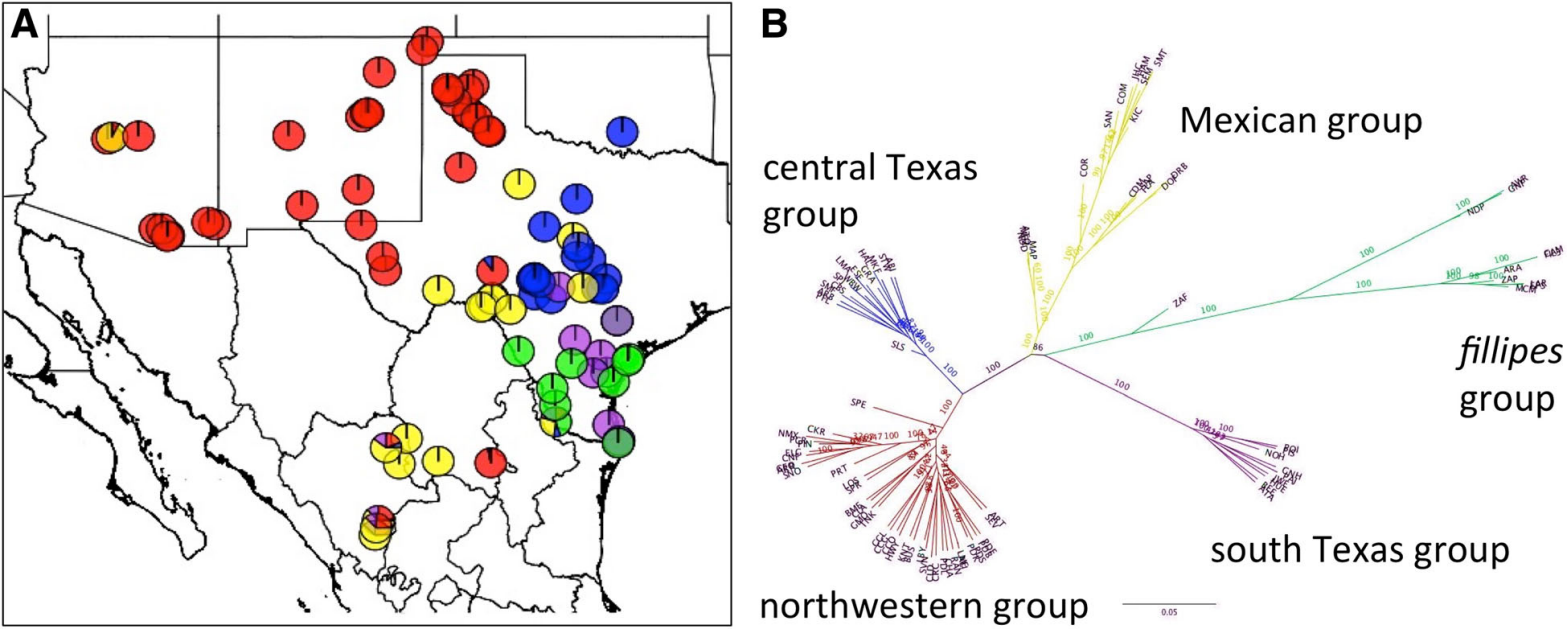
Population structure of 89 *P.hallii* individuals. **a** Geographic sampling locations colored by proportion of STRUCTURE group membership. **b** RAxML phylogenetic tree. Major clades on the tree are colored to match the corresponding groups in (**a**)

**Fig. 2:**
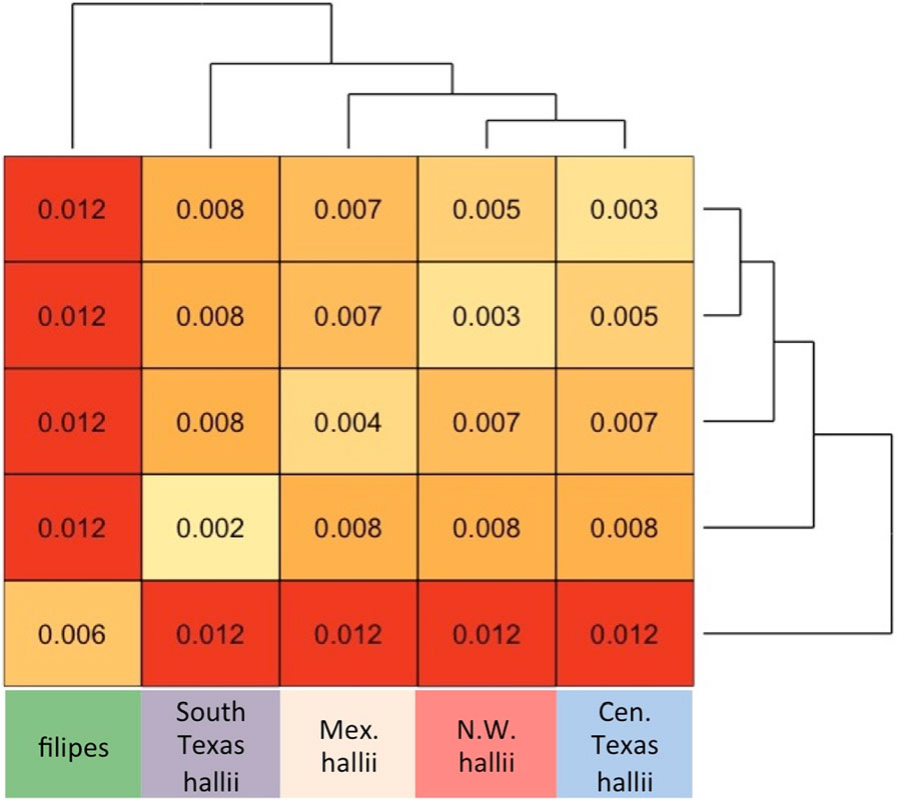
The average number of pairwise nucleotide differences between *P.hallii* subgroups . Differences between groups are shown on the off-diagonal (D_xy_) and differences within groups (**π**) are shown on the diagonal. Groups correspond to STRUCTURE groupings

Genome sequencing of *P. hallii* has revealed that much of the large pericentromeric region of the genome consists of a low-recombination region with a high level of repetitive DNA. Such large pericentromeric regions are common across plant species [17, 18]. To reduce false SNP calls in these regions, we eliminated areas of low sequencing/mapping depth (<8 reads) and unusually high heterozygosity (see Methods). After filtering, we identified 9.2 million probable SNPs across the 89 diverse *P. hallii* accessions. Given that an appreciable rate of erroneous variant calls likely still occurs in the data, we estimated that the upper bound for pairwise diversity (π), based on callable sites (~ 46% of the genome), as 0.008 within var. *filipes* and 0.007 within *var. hallii*. Further, pairwise divergence (D_xy_) was greater for comparisons between *var. filipes* and any *var. hallii* groups than between any of the *var. hallii* groups (Figs. 1, 2).

The distribution of SNP variation confirmed a strong population split between *var. hallii* (*N* = 78) and *var. filipes* (*N* = 11). Within *var. hallii*, linkage disequilibrium decayed (*r*^*2*^ < = 0.2) at around 6 kb while within *var. filipes*, linkage remained high (*r*^*2*^ > 0.3) at distances greater than 25 kb (Additional file 1: Figure S1). We hypothesize that greater linkage disequilibrium in *var. filipes* may result from a small effective population size and possible historical population bottlenecks. In addition to the predicted divergence between subspecies, analysis with both STRUCTURE and RAxML revealed at least four major groups within *var. hallii* (Fig. 1, Additional file 1: Figure S2). The four major groups within *var. hallii* included a widespread group that occupies northwestern Texas westward into New Mexico and Arizona (red), and three more geographically overlapping groups. A small group is restricted to central Texas (blue) that overlaps with a second group that ranges from central Texas south into northern Mexico (yellow). A third *var. hallii* subgroup occurs in south Texas (purple) and overlaps to a great extent with the range of *var. filipes*. Interestingly, the degree of divergence between *P.hallii* var. *hallii* subgroups (max *D*_*xy*_ = 0.008), which are not formally recognized as subspecies, approached the level of divergence found between recognized subspecies var. *filipes* and var. *hallii* (*D*_*xy*_ = 0.012, Fig. 2).

We found evidence that hybridization occurs between *var. filipes* and *var. hallii* albeit at a relatively low rate. If hybridization between subspecies were common throughout history we would expect many individuals to show evidence of mixed ancestry however only a single individual located in the southern part of the *var. filipes* range showed evidence of possible admixture between *var. filipes* and the Mexican *var. hallii* group (Fig. 1). A sympatric population between *var. filipes* and *var. hallii* has been identified near Gonzalez, TX. The resequenced *var. filipes* and *var. hallii* lines from this population showed no evidence of mixed ancestry. In contrast, several individuals showed evidence of hybridization between groups within var. *hallii*. Seven of 78 *var. hallii* individuals showed significant evidence of mixed ancestry between one or more groups. All of these mixed individuals were collected from the southern part of the range (Fig. 1).

**Fig. 3:**
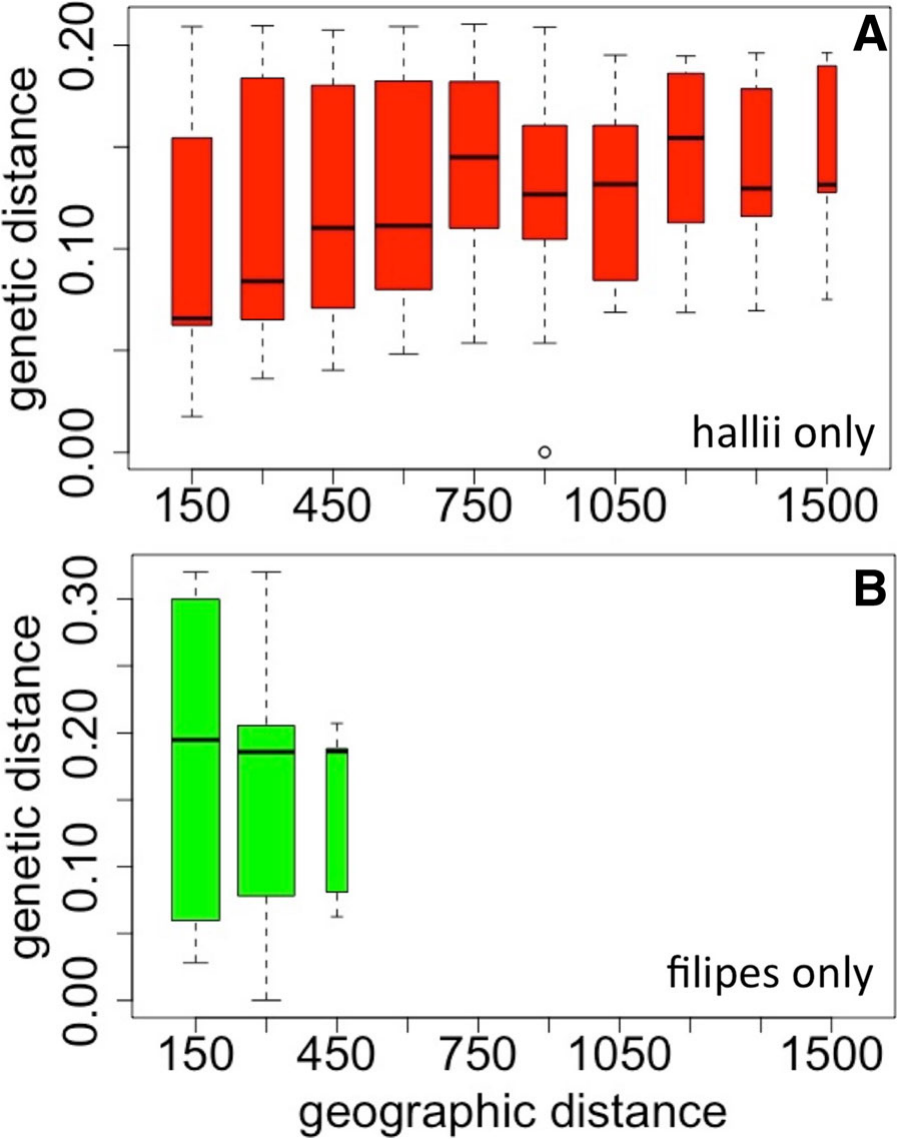
Isolation by distance. Genetic distance between pairs of plants binned by geographic distance (in km). **a** var. *hallii* only, **b** var. *filipes* only

Geography is correlated with genetic distance across the range of *P.hallii* (Fig. 3). Correlation of genetic distance with geographic distance can arise due to limited interbreeding between geographically separated groups as well as due to hierarchical population structure [19]. In *P.halii* we found that overall isolation by geographic distance (IBD) was not significant across the entire *P. hallii* collection after controlling for the effects of population substructure (see Methods). However, IBD was strong and statistically significant within three of the individual subgroups: Northwestern hallii (Fig. 1, red), Mexican hallii (yellow), and South Texas hallii (purple) (Additional file 2: Table S5). Although the NW hallii group spans the greatest geographic distance, IBD was less strong in this group (Mantel’s *r* = 0.580) than within the smaller South Texas group (Mantel’s *r* = 0.728). We hypothesize that genetic diversity across *P.hallii* is the result of some isolation by distance combined with hierarchical structure that developed as a result of expansion from southern glacial refugia following the last glacial maximum.

### Climate

We examined variation in precipitation and temperature across the species range using data from 19 Bioclimatic variables from the WorldClim database (Additional file 1: Figures S3 and S4). The major contrasting axes of variation in precipitation across the range were most strongly correlated with annual mean precipitation, seasonality of precipitation, and mean precipitation in the warmest quarter (Additional file 1: Figure S3). Major variation in temperature was characterized most strongly by variation in mean daily temperature range, isothermality, and minimum temperature of the coldest month. Aridity, a measure of water stress imposed on plants due to the combined influence of solar radiation, precipitation and temperature, also varied significantly across the range. In general, climate in the study area varies across a northwest to southeast axis, from the hot dry deserts of Arizona, New Mexico, western Texas, and northern Mexico to the more mesic regions along the Gulf Coast (Additional file 1: Figure S5). *Var. filipes* is found only in the coastal part of the range in South Texas, whereas *var. hallii* is found throughout the study area with the exception of some parts of the Gulf Coast region (Fig. 1).

Population structure within *P. hallii* as a whole was correlated with many of the major climate variables we examined. Genetic distance between individuals, regardless of subspecies identity, was significantly correlated with differences in collection site climate including mean annual precipitation, precipitation seasonality, average precipitation in the warmest quarter, mean daily temperature range, minimum temperature of the coldest month, and aridity (all *P* < 0.01, Table 1). Correlation of climate with genetic distance between individuals was strongest for minimum temperature and temperature daily range (both Mantel’s *r* = 0.42).

**Table 1:** Associations between climate variables and genetic variation

Climate Variable	Description	range	Correlation with genetic distance (Mantel’s r)	No. Associated SNPs	No. SNPs in genes	No. Genes Affected^a^
BioClim3	isothermality (daily/annual temperature range)	0.017–0.029	−0.04	51	32*	26
BioClim2	mean daily temperature range (degrees × 10)	84–195	0.41**	32	10	7
BioClim6	minimum temperature of the coldest month (degrees C)	2.20–5.32	0.42*	12	12*	5
BioClim12	annual mean precipitation (mm)	220–953	0.22**	13	6	4
BioClim18	precipitation in the warmest quarter (mm)	99–271	0.20*	1	1	1
Aridity	mean annual precipitation/mean annual potential evapo-transpiration	0.128–0.706	0.24*	42	6*	4

^a^The number of unique genes containing the snps associated with a climate variable

* *p* < 0.05; **p< 0.01

### Climate GWAS

We conducted a genome-wide association study (GWAS) of high frequency (MAF > 10%) SNPs against each major climate variable (Additional file 1: Figure S5). We repeated the analysis for all *P. hallii* individuals and for the set of only *var. hallii* plants (Additional file 1: Figure S6). We used the STRUCTURE population membership coefficients as a control for the correlation between climate and relatedness (see Methods). This method provided the strongest control for correlations between population structure and temperature variables with lesser control of correlations with precipitation (Additional file 1: Figure S5). Using Bonferroni corrected significance cut-offs we identified 139 unique SNPs significantly associated with climate across all plants and 170 SNPs associated with climate within *var. hallii* alone (Table 1). In both analyses the largest number of SNPs were associated with isothermality (*N* = 51 and 137, respectively). While 41 SNPs were associated with aridity across the combined group, there were no SNPs associated with aridity in var. *hallii* alone, indicating this association may be primarily driven by differences between subspecies. Many climate associated SNPs in both analyses fell within genic or 1 kb promoter regions of the genome. In the analysis of all plants, for at least three climate variables - isothermality, minimum temperature of the coldest month, and aridity – associated SNPs were more often found in genes than expected by chance (*p* < 0.05, Table 1). In addition to falling within genic regions, the climate-associated SNPs were also weakly enriched for non-synonymous substitutions (*p* = 0.04) suggesting many may be functional in nature. To test for the effect of recent strong selection on genes containing climate-associated variants we calculated both variable-site nucleotide diversity (π_var_) and Tajima’s D. We found no evidence of reduced diversity or an excess of rare variants in climate-associated genes. Variable site π was similar in climate and non-climate associated genes (π_var_ = 0.163 and 0.161, respectively). Tajima’s D was close to zero and similar between the gene groups (D = − 0.17 and − 0.21, respectively).

Because there are strong correlations between climate and geography across the study area, we examined more closely how both factors influence variation in the candidate climate SNPs identified by GWAS. We used redundancy analysis (RDA) to calculate the proportion of variation in each set of candidate SNPs (for each climate variable) that can be explained separately by climate and by geography (Additional file 2: Table S4). For all sets of candidate SNPs the variation explained by the combination of climate and geography combined was greater than expected by chance (permutation tests *p* < 0.001, Additional file 2: Table S4). For all sets of candidate SNPs, a greater percentage of variation could be explained by climate than by geography. Variation in minimum temperature of the coldest month (BioClim variable 6) explained the greatest percentage of variation in candidate SNPs (41.9%, *n* = 12 SNPs). Geography explained only 5.1% of variation in the same SNP set. Most other climate variables including mean daily temperature, isothermality, annual precipitation and aridity explained between 12 and 15% of candidate SNP variation, geography explaining between 5 and 11% of variation (Additional file 2: Table S4).

**Fig. 4:**
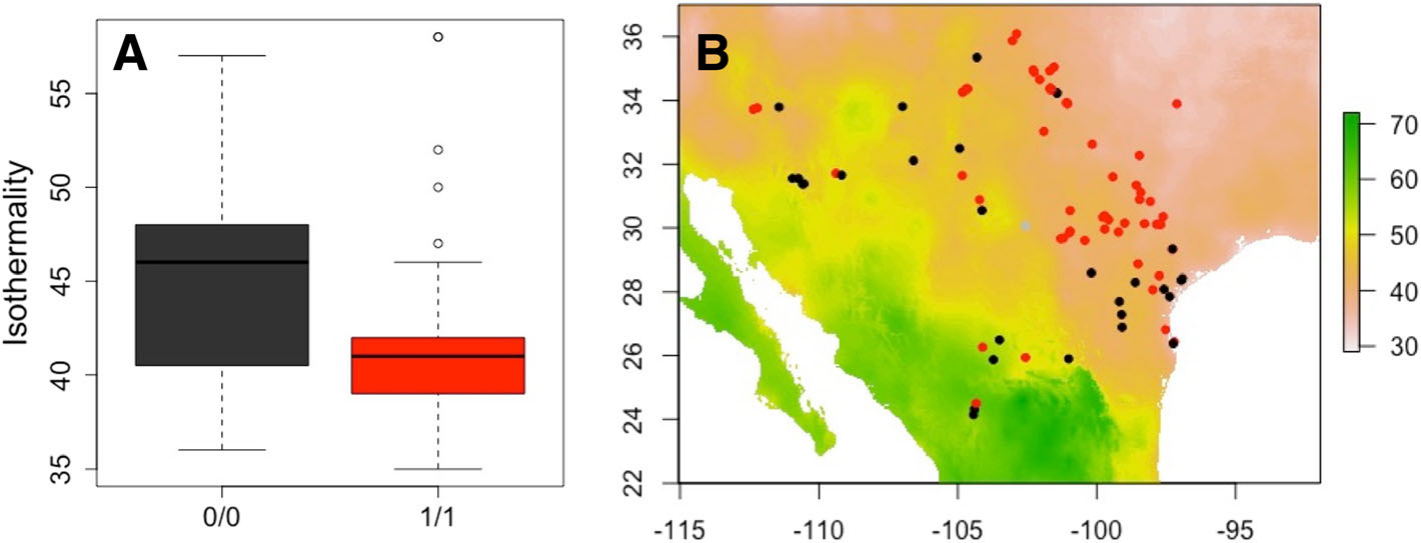
Distribution of genotypes at a SNP in candidate gene Pahal.D02753 that is putatively involved in drought recovery and is associated with climate isothermality (daily/annual temperature range). **a** Home site isothermality values for homozygous genotypes at the SNP. **b** Geographic distribution of SNP genotypes. Map shading represents isothermality ratios. Homozygous reference allele in black, homozygous alternate allele in red, missing genotype in grey

Following genome-wide analyses, we examined the putative function of genes containing climate associated SNPs (Additional file 2: Tables S1 and S2). SNPs associated with aridity were in genes enriched for functions related to dormancy processes, seed maturation, response to hyperosmosis and salinity, and abscisic acid metabolism and biosynthesis. One SNP in Pahal.C02265, a gene associated with regulation of stomatal closure and transpiration in *Arabidopsis* [20], was correlated with both aridity and annual precipitation but fell just above the *p*-value cutoff used in this study (*p* = 2.0 × 10^− 8^ for aridity and *p* = 2.3 × 10^− 8^ for annual precipitation). SNPs in genes associated with daily temperature range were enriched for functions related to hormone metabolism and biosynthesis. Lastly, SNPs associated with isothermality were enriched for involvement in drought recovery. An isothermality associated SNP fell within Pahal.D02753. The *Arabidopsis* ortholog of this gene (AT1G70670) is a negative regulator in abscisic acid (ABA) signaling (Fig. 4; [21]).

## Discussion

In this study, we were able to characterize genomic variation across geographic space for 89 accessions of the C4 perennial grass species, *P. hallii*. Consistent with a previous smaller-scale study [11], we found strong patterns of population structure between the two previously identified subspecies (varieties) in this system, *var. hallii* and *var. filipes*. We also found evidence that *var. hallii* is further partitioned into at least four other groups, which all have partially overlapping geographic ranges. Each of those groups may constitute a different ecotype (as defined by [6]), which may have evolved allopatrically and only recently come into secondary contact. To explore the extent to which climate and geography have shaped genomic variation in this species we used whole genome sequencing to assay over 9 million SNPs and identified many which are strongly associated with climate variation. We discuss these findings in more detail below.

### The structure of species complexes

Population structure analysis of *P. hallii* revealed that it is partitioned into at least five subgroups. This revelation of complexity of population structure in *P. hallii* parallels recent findings in its close relative switchgrass (*P. virgatum*). The literature on switchgrass typically partitioned that species into upland and lowland ecotypes [22, 23], which have similar morphological differences as between *var. hallii* and *var. filipes*. However, just as in this study recent population structure analyses and new collections in the southern US have revealed that other major groups also exist within *P. virgatum* [12, 24].

Overall, the divided population structure of *P. hallii* and other plant species suggests that many widespread species exist as subspecies complexes, with groups at different stages of the speciation continuum occurring across their range [25]. The most divergent subgroups, *var. hallii* and *var. filipes*, are at least partially reproductively isolated. This idea is supported by Lowry et al. [15] who identified a two-locus Dobzhansky-Muller incompatibility in a cross between *var. hallii* and *var. filipes*. There may also be some reproductive isolation between the four subgroups identified within *P. hallii* in this study. Admixture between the varieties appears to occur at a relatively low rate, despite having overlapping geographic ranges. Future studies should quantify reproductive isolating barriers between these groups and sample more individuals within populations, especially those in sympatric regions, to establish the extent of gene flow between the various groups.

The patterns of population structure of *P. hallii* may also provide insights into general biogeographic divisions across its range. In particular, there is a strong break in population structure that runs diagonal through West Texas from northeast to southwest, (a break dividing the red and yellow population subgroups in Fig. 1a). The break is strongly associated with a transition from lower elevation in the east to higher elevation in the west. To the west of the break are the higher elevation populations of *P. hallii*, which primarily cluster as a single distinct group. Lowry et al. [11] found that the morphology of these high elevation populations is also distinct from the rest of *P. hallii*, as these plants have a larger overall size and have the largest seeds of any of the plants in the species complex. To the east of the break are all the other ecotypes of *P. hallii*, including *var. filipes*. This break in Texas corresponds to the transition from the gently rolling Edward/Stockton Plateaus to mountainous Trans-Pecos region in the south and the transition between the rolling lower plains to the high plains further north. These vegetation regions of Texas are well established [26, 27]. While few molecular population structure studies of plants have been conducted in this region, a recent study found a very similar ecogeographic split in *Melampodium leucanthum* in West Texas [28]. Multiple population genetic groups have also been identified in animals in this region, with the origins of these groups hypothesized to be the result of multiple Pleistocene refugia [29, 30].

The extent to which the geographic ranges of subgroups of *P. hallii* overlap may actually be underestimated by this study. In particular, the “Mexico” subgroup (Fig. 1, yellow) may be more widespread. One accession collected from a population in Arizona (COR) clustered with populations from central Texas and Mexico, just as it did in Lowry et al. [11]. The population exists at a relatively low elevation (1110 m.a.s.l.) compared to other collections in Arizona, New Mexico, and West Texas. This suggests the possibility that the Mexico subgroup is also distributed throughout the lower elevations of the desert Southwest of the United States.

### Climate associations

Similar to studies of other plants, there is evidence that genetic distance is correlated with both geographic separation and major climate variables in this species. Despite confounding between climate and geography we were able to identify SNPs that were significantly associated with all major climate variables. These in turn are likely to be driving phenotypic variation that is correlated with environment. While variation in climate-associated SNPs was also associated with geography, more candidate SNP variation could be explained by climate than by geography in all cases.

It is unclear whether climate or isolation by distance has a stronger influence on genome-wide genetic similarity in natural plant populations, but at least one study in *Arabidopsis* [31] demonstrated an equal influence of both factors. We find climate associated SNPs are not often associated with more than one climate variable in *P. hallii*. This result parallels findings of Fournier-Level et al. [32], who found that while fitness associated SNPs in *Arabidopsis* had non-random distributions, they were not often associated with fitness in more than one environment.

In *P. hallii* climate associated SNPs were more often found in genes than expected by chance, which is similar to findings in [31, 33]. This suggests that the candidate genes we identified contain functional or regulatory changes that contribute to climate adaptive phenotypic differences between population groups. Genes containing climate-associated variants were only weakly enriched for non-synonymous changes, which contrasts somewhat with recent findings in natural populations of other species. We also found little evidence that genes containing climate associated SNPs have been under stronger directional or purifying selection than other genes in the genome. Climate SNPS also do not appear to have been subject to recent selective sweeps. In *Arabidopsis* and cultivated *Sorghum*, climate associated SNPs were often non-synonymous [31, 34] and in *Brachypodium*, more than half of climate-associated loci showed signs of recent selection [35].

The putative function of many loci associated with climate variation in *P. hallii* are linked to adaptive traits that are known or suspected to vary between locally adapted populations. Genes associated with both dormancy and response to hyperosmosis and salinity (both experienced during drought stress) contained climate associated SNPs. *P. hallii* is exposed to variable levels of drought stress throughout its range during the summer and becomes dormant in the winter months. Population subgroups, perhaps nascent ecotypes, would be predicted to have appreciable differences in both drought tolerance and avoidance response (dormancy). One candidate gene (Pahal.D02753) containing climate-associated variation is likely to be involved broadly with drought recovery through the regulation of the key stress hormone ABA.

## Conclusions

Using population genomic sequencing and analyses we identified multiple population subgroups within the perennial C4 grass *P.hallii*. These population subgroups were partially overlapping across the species range and may represent early stages in the formation of subspecific ecotypes. Genomic variation across subgroups was significantly driven by adaptation to broad-scale climate variation in temperature, precipitation and aridity. Genes containing climate-associated SNPs most likely function in stress response regulation, tolerance, and recovery. These genes provide targets for further research into the molecular basis of adaptation along ecological gradients, particularly in perennial grass species.

## Methods

### Plant collections and genome sequencing

Many of the accessions used in this study were collected from field sites as seeds or live plants in the years 2010–2014. The authors thank the following for providing seed collections and permission for our collections: The Ladybird Johnson Wildflower Center, The Brackenridge Field Laboratory, The Nature Conservancy of Texas, The State Parks of Texas, The Kika de la Garza Plant Materials Center, members of Christine Hawkes laboratory, and The University of Arizona and Arizona State University Herbaria. Seeds were collected from private lands through permission granted by the Texas Ecolab project. Seeds were imported from Mexico by under a University of Texas herbarium permit, number SGPA/DGVS/04170/14.

A single accession was sampled from each population to maximize sampling across the geographic range of *P. hallii* and to avoid biases introduced by strong pairwise among deme population structure [36]. Because only a single plant was sampled at each location, in this study we can draw inferences in relation only to range-wide patterns of genetic diversity and correlations with climate but not about within population variation in allele frequencies. Additional accessions were acquired from various existing seed collections (Additional file 2: Table S3). Collections were processed as described in Lowry et al. [11]. All accessions that were used in this study were grown from seed in the University of Texas at Austin greenhouses. High-quality DNA was extracted from leaf tissue using Qiagen DNeasy Plant Mini Kits (Qiagen, Hilden, Germany).

Whole genome sequencing was performed at two sequencing centers. Eighty lines were sequenced at the Joint Genome Institute (Walnut Creek, CA) on either the Illumina HiSeq2000 or HiSeq2500 platforms. The remaining libraries were sequenced at the University of Texas at Austin. Sequencing was paired-end at 150 bp read length.

Short read data has been archived in the NCBI Short Read Archive (https://www.ncbi.nlm.nih.gov/sra). Accession numbers are listed in Additional file 2: Table S3.

### SNP calling and filtering

Reads were aligned against the v2 draft *P.hallii* genome (*filipes* variety; FIL2 genotype, available at phytozome.org) using BWA MEM (v0.7.12) and the parameters (−M –p –t8). Before alignment, the genome was repeat masked for 24-mers occurring >= 650 times. This masked approximately 5% of the genome. BAM files were screened for PCR duplicates and areas around indels were realigned using GATK IndelRealigner [37]. SNPs were called on the filtered alignments using the VarScan v2.4.1 mpileup2snp command [38] with a minimum coverage of eight reads and a minimum supporting read count of four to call SNPs. All SNPs within 25 bp of a repeat masked region or a gap were eliminated from the data set to eliminate low-quality SNP calls. Heterozygous calls were refined using a binomial exact test of read counts for each allele, with heterozygous calls excluded at a combined significance level of *p* > 0.05. SNPs were only retained that had a greater than 90% call rate across all individuals and that had at least two individuals supporting each homozygous allele.

The reference genome line (FIL2) was included in the original sample set, and as a conservative control for sequencing or assembly errors calls at any site where an alternate allele was called in the re-sequenced FIL2 line were considered missing data (*N* = 15,714 nucleotide positions). We also observed unusually high numbers of heterozygous calls in pericentromeric regions of the genome. This is likely due to inaccuracy of alignment in these areas, which are known to often be repeat-rich in plants [17, 18]. The observed level of heterozygosity in *P. hallii* at microsatellite markers was about 5% [11], so we eliminated all SNP calls with greater than 5% heterozygous calls. We also eliminated SNPs with quality scores less than 40, greater than 10% missing genotype calls, more than two alleles, and greater than + 2 standard deviations read coverage (103 reads). The final data set contained 9,270,046 SNPs genotyped across 89 individuals. SNPs were annotated using SNPeff v4.1 k [39] and the v2.0 annotation of the *P. hallii* genome (www.phytozome.org).

### Analysis of population structure and linkage

A set of synonymous SNPs were isolated for use in analyses of population structure. Using VCFtools v0.1.11 [40], we generated a set of synonymous variants that occurred only on major scaffolds and were no closer to one another than 3000 bp (*N* = 28,640) to control for possible linkage disequilibrium. We designate this set of markers as ‘neutral SNPs’.

We analyzed the neutral SNPs with RAxML v8.0.6 [41] to generate an un-rooted phylogenetic tree reflecting the most likely relationships between individuals. Neutral SNPs were concatenated for each individual. We used a tree-building model with no rate heterogeneity and a correction for ascertainment bias (ASC_GTRCAT), with a maximum of 1000 bootstrap replicates. The resulting tree was visualized using FigTree (http://tree.bio.ed.ac.uk/software/).

The neutral SNPs were also used to analyze population clustering with STRUCTURE [42]. We converted the SNP data to STRUCTURE format using the program PGDSpider [43] and then completed five runs each for values of *K* = 2–8. Each run had a default 10,000 burn-in MCMC iterations and an additional 10,000 iterations following. Results for all runs were summarized using Structure Harvester [44] and plotted with Distruct [45]. The number of structured groups with the highest deltaK value [46] was 3. However, because the RAxML analyses identified at least five major clusters, we used five population clusters for downstream analyses.

Isolation by distance (IBD) was tested using a Mantel test, in the R package vegan, of the matrix of geographic distances between sampling locations vs. the matrix of genetic distances between individuals. Genetic distance between individuals was calculated as 1-(probability of identity by state) for neutral SNPs in TASSEL [47]. Geographic distances between sampling coordinates were calculated using the R package Imap v1.32 [48] . Significance of IBD was tested using 999 permutations stratified by subpopulation group membership. We also used Mantel tests to evaluate correlations between pairwise differences in collection site climate variables and pairwise genetic relatedness.

### Climate association analysis

Full BioClim environmental data (19 variables) was downloaded for the study area from http://www.worldclim.org/bioclim. Climate variable values were extracted for a set of 127 *P.hallii* known sampling locations using the data portal at http://dataportal-senckenberg.de/dataExtractTool/. To choose climate variables for association analysis, we used principal components analysis to determine the axes of greatest climate variation across the study area and to identify variables with the least amount of correlation. First, we determined the principal components of the set of all precipitation variables (bio12-bio19) and identified the individual variables that loaded most heavily on the first three principal components (Additional file 1: Figure S3). The variables of interest were bio12 (annual mean precipitation), bio15 (precipitation seasonality), and bio18 (amount of precipitation in the warmest quarter). Due to missing data values for a subset of the collection sites, bio15 was later dropped from the analysis. Second, we determined the principal components of the set of all temperature variables for the study area (bio1-bio11). The variables that loaded most heavily on the first three components were bio2 (temperature mean daily range), bio3 (isothermality = daily/annual range), and bio6 (minimum temperature of the coldest month). We also downloaded data on aridity for the region from the CGIAR-CSI global database (http://www.cgiar-csi.org/data/global-aridity-and-pet-database). Aridity is calculated as mean annual precipitation divided by mean annual potential evapo-transpiration. Data for climate variables isothermality, minimum temperature of the coldest month, and precipitation in the warmest quarter were transformed to improve normality before analysis.

We used TASSEL to test the association between SNPs and each selected climate variable. To avoid bias we only analyzed SNPs with a minor allele frequency (MAF) of 10% or greater (*N* = 3,059,501). To control for population structure, we tested three linear models: one that incorporated a random effect kinship matrix, one that used the STRUCTURE population membership covariates (*Q* values), and one that used both [49]. Use of both Q coefficients and a kinship matrix together produced conservative results that over-corrected for population structure, evident as skewed *p*-value distributions. Because use of a model with Q coefficients alone provided stronger control for structure than using a K-matrix alone, we chose the former for all subsequent analyses. We used a Bonferroni corrected significance threshold of *p* < 1.63 × 10^− 8^ for associations. Enrichments for SNP position (genic/non-genic) and type (synonymous/non) were conducted using Fisher exact tests or the chi-squared approximation in R. We repeated the association tests within *var. hallii* alone, excluding all *var. filipes* individuals. In that analysis, there were 2,241,150 SNPs with MAF > 10%. We used a Bonferroni corrected significance threshold of *p* < 2.23 × 10^^− 8^.

We tested whether regions of potentially low-quality alignment and SNP calling in peri-centromeric regions of the genome were likely to generate false positives. We used TASSEL to reanalyze only SNPs that fell outside of peri-centromeric regions. In general, climate associated SNPs in that analysis were not more or less often located in genic regions than for the full analysis. Because this is an indicator that SNP calling errors do not strongly contribute to error in the identification of climate associated SNPs, we present here results for SNPs in all regions of the genome.

#### Redundancy analysis (RDA)

RDA was performed on the set of significant candidate SNPs associated with each climate variable. To test the influence of geography on each candidate SNP set we used the first principal component (PC) of latitude and longitude of all plant collection sites as a predictor variable. Missing genotype values were imputed as heterozygous. Significance of each constrained axis of variation (climate variable and geographic PC) was measured using 999 permutation tests. RDA was executed using the vegan package in R [50]. For the single SNP associated with BioClim variable 18, we instead used linear regression of SNP variation on climate and geographic PC separately to measure the amount of variation explained as R^2^ values.

### GO term enrichment analysis

We tested for enrichment of gene-ontology (GO) terms among associated SNPs for each climate variable. To annotate GO terms, all *P.hallii* reference proteins were paired with their best *Arabidopsis* protein matches using BLAST (blastp) at an *e*-value cutoff of 10^− 3^. This set of proteins (including duplicates) was used as the background gene set against which to test hypotheses of significant enrichment of GO terms for each gene set of interest. Subsequent GO enrichment tests for genes near climate associated SNPs were done using the R BIOCONDUCTOR package topGO [51]. We used the Bioconductor database ‘org.At.tair.db’ for annotation and the algorithm ‘classic’ (statistic = ‘fisher’) for statistical tests. We report GO terms with uncorrected enrichment *P*-values <= 0.05 (Additional file 2: Table S1).

## Additional files

Additional file 1: **Figure S1.** Linkage vs. physical distance (bp) between markers in the genome. **Figure S2.** Plots of STRUCTURE membership at various k values. **Figure S3.** Analysis of climate variation via principal components. **Figure S4.** Heatmap of 19 Bioclim variables by collection site. **Figure S5.** Maps of variation in climate variables across the range of *P.hallii.* **Figure S6.** Manhattan plots of SNP association with climate variables for *P.hallii*. (PDF 1693 kb)

Additional file 2: **Table S1.** GO terms significantly associated with each climate variable. **Table S2.** Candidate genes containing climate associated SNPs. **Table S3.** Collection locations and SRA accession numbers for sequenced plants. **Table S4.** RDA analysis of SNP variation. **Table S5.** Analysis of isolation by distance. (XLSX 26 kb)

## Abbreviations

ABA: Abscisic acid
BAM: Binary alignment
BioClim: Bioclimatic variables derived from monthly temperature and rainfall values in the WorldClim database (http://www.worldclim.org/bioclim)
FIL2: Filipes line 2, the reference genome accession
GATK: Genome analysis toolkit (software, [37])
GWAS: Genome wide association study
IBD: Isolation by distance
MAF: Minor allele frequency
MCMC: Markov Chain Monte Carlo
QTL: Quantitative trait locus
RAxML: Randomized Axcelerated Maximum Likelihood (software, [41])
RDA: Redundancy analysis
SNP: Single nucleotide polymorphism
TASSEL: Trait Analysis by aSSociation, Evolution and Linkage (software, [47])
TX: Texas
