# Supplementary material for: Population genomics and climate adaptation of a C4 perennial grass, Panicum hallii (Poaceae)
Source: BMC Genomics. 2018 Nov 1;19:792. doi: 10.1186/s12864-018-5179-7 (PMC6211516; doi:10.1186/s12864-018-5179-7)
Supplement: Supplementary file 1 — Figure S1. Linkage vs. physical distance (bp) between markers in the genome. Figure S2. Plots of STRUCTURE membership at various k values. Figure S3. Analysis of climate variation via principal components. Figure S4. Heatmap of 19 Bioclim variables by collection site. Figure S5. Maps of variation in climate variables across the range of P.hallii. Figure S6. Manhattan plots of SNP association with climate variables for P.hallii. (PDF 1693 kb) [file 12864_2018_5179_MOESM1_ESM.pdf]

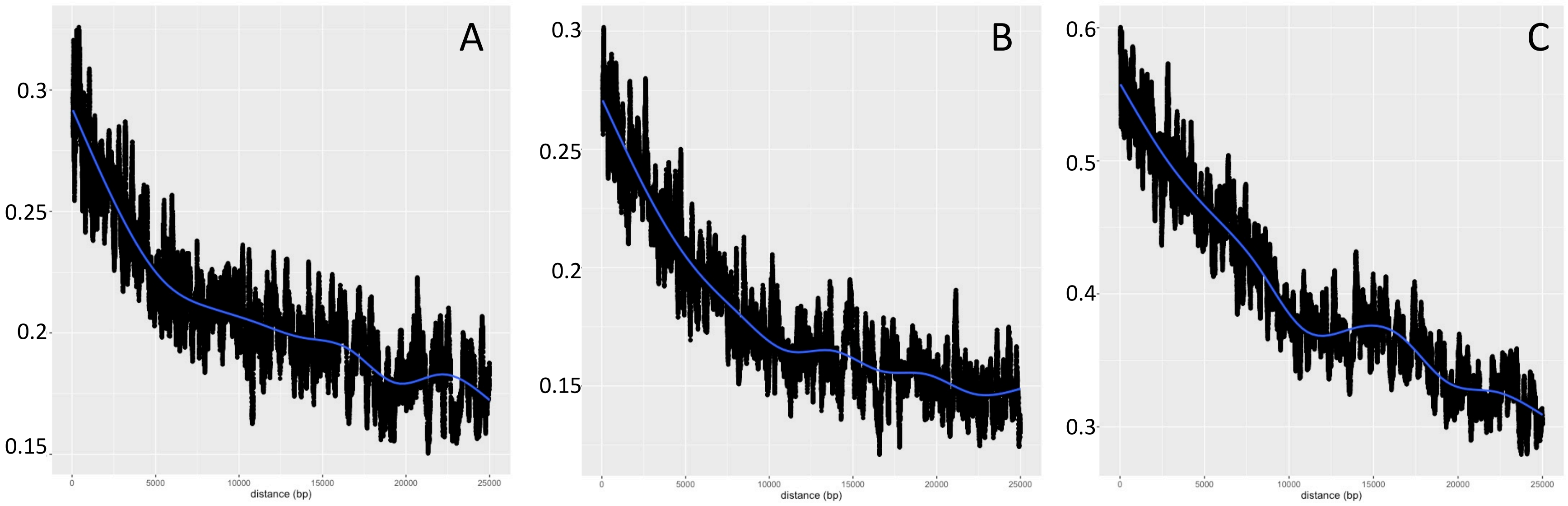

Fig. S1. Linkage (r^2) vs. physical distance (bp) between markers. Data are taken from chromosome 1. A) All *P.hallii* individuals. B) P.*hallii* var. *hallii* individuals only (N=78). C) *P.hallii* var. *filipes* individuals only (N=11). Note, distances on the x-axes are the same between plots but linkage on the y-axes differ between plots.

Fig. S2. STRUCTURE plots.

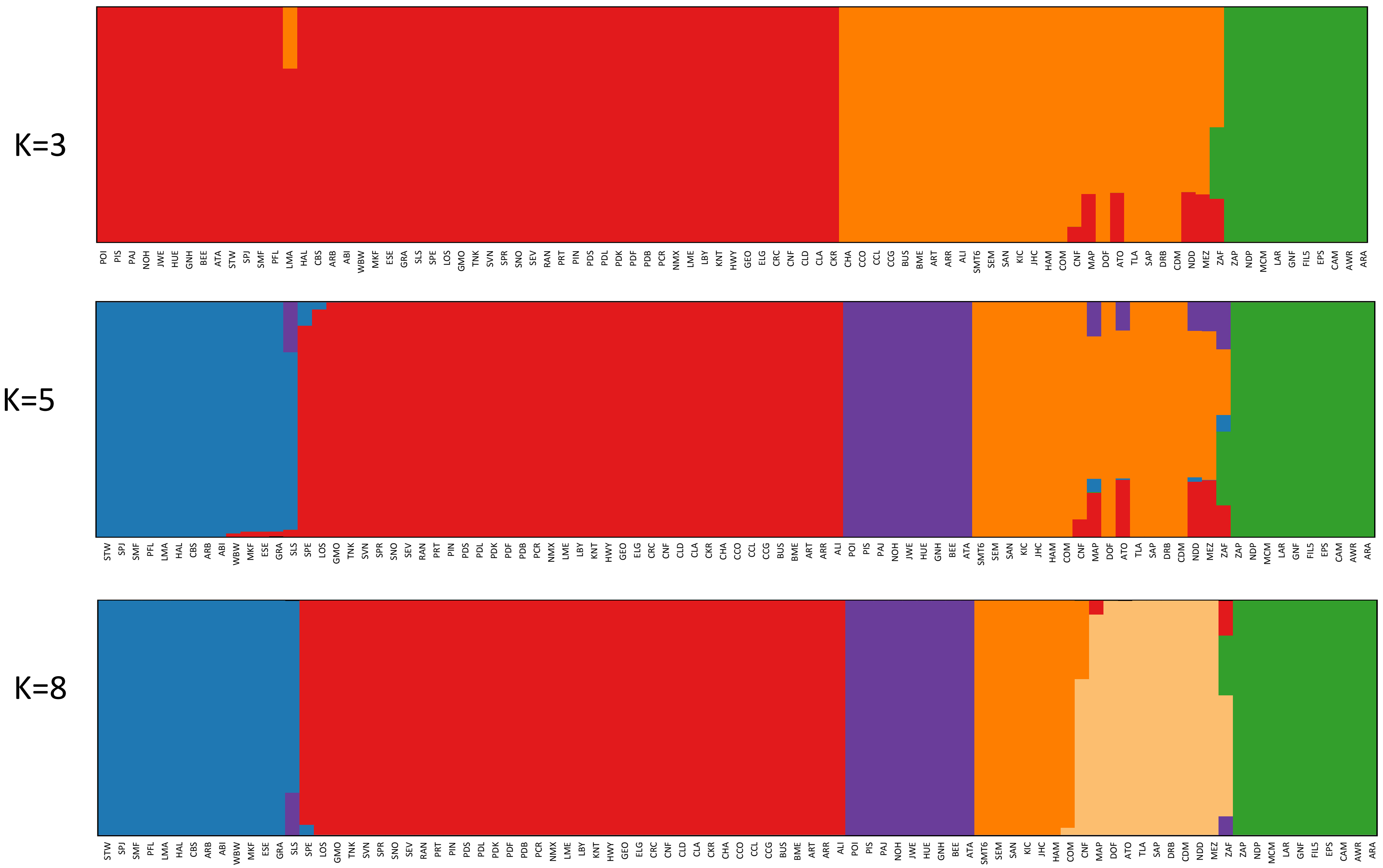

Fig S3. Analysis of climate variation via principal components. A) PCA of precipitation related Bioclim variables. B) PCA of temperature related Bioclim variables. Plots and tables show the loadings of each variable onto the first 2-3 components. Loadings less than abs(0.1) in the tables are not shown. Points in the plots represent component values for *P.hallii* sampling sites. The variables chosen for association analysis are in bold.

A

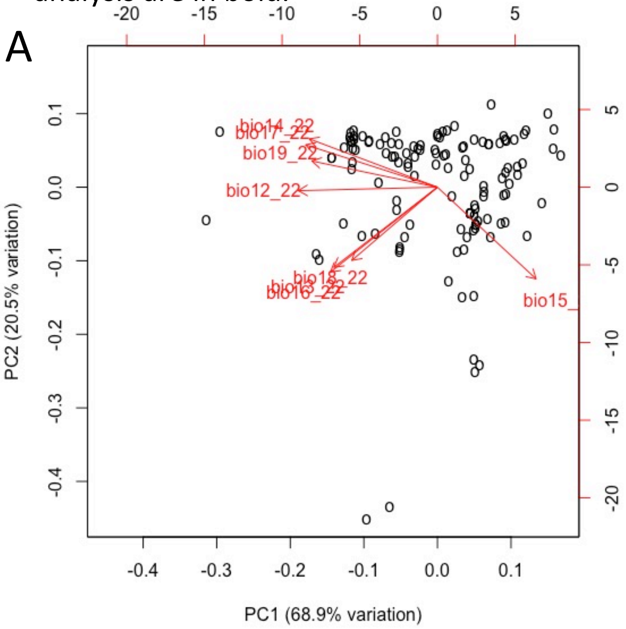

**Precipitation**	var	Comp.1	Comp.2	Comp.3
**annual mean precip.**	**bio12_22**	**-0.423**		
precip. wettest month	bio13_22	-0.315	-0.446	0.431
precip. driest month	bio14_22	-0.39	0.269	
**precip. seasonality**	**bio15_22**	0.3	**-0.511**	0.163
precip. wettest quarter	bio16_22	-0.325	-0.473	0.195
precip. driest quarter	bio17_22	-0.401	0.236	
**precip. warmest quarter**	**bio18_22**	-0.26	-0.408	**-0.846**
precip. precip. coldest quarter	bio19_22	-0.382	0.146	0.161

B

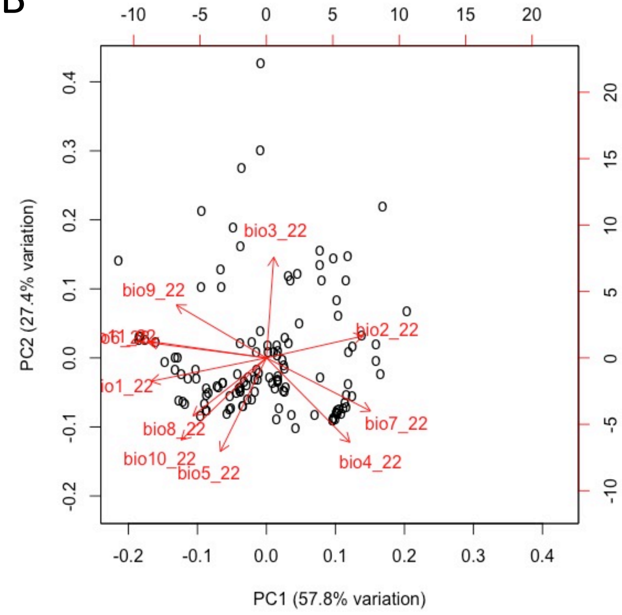

**Temperature**	var	Comp.1	Comp.2	Comp.3
temp. warmest quarter	bio10_22	-0.282	-0.395	
temp. coldest quarter	bio11_22	-0.388		-0.106
annual mean temp.	bio1_22	-0.382	-0.113	
**mean daily range**	**bio2_22**	0.32	0.105	**-0.575**
**isothermality**	**bio3_22**		**0.484**	-0.539
seasonality	bio4_22	0.276	-0.406	
max. temp. warmest month	bio5_22	-0.152	-0.45	-0.436
**min. temp. coldest month**	**bio6_22**	**-0.392**		
annual range	bio7_22	0.345	-0.256	-0.215
temp. wettest quarter	bio8_22	-0.243	-0.278	-0.311
temp. driest quarter	bio9_22	-0.299	0.255	-0.135

Fig. S4. Heatmap of 19 Bioclim variables by collection site. Variables have been converted to z-scores. Variables used for association analysis are marked with an asterisk. Columns represent collected individuals, grouped by major STRUCTURE group membership, colored as in Fig. 1.

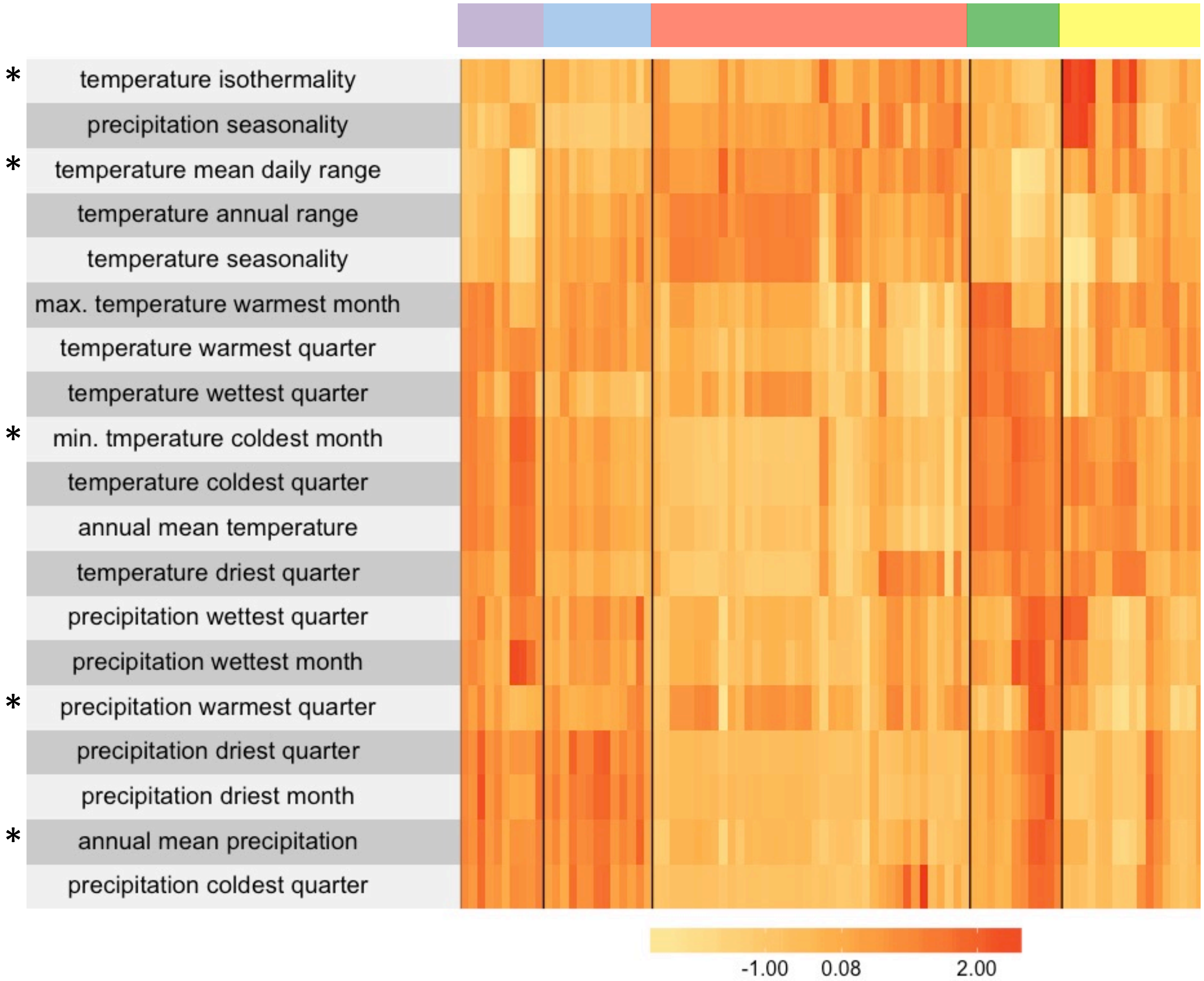

Fig. S5. *P.hallii* range maps with values for each of 5 climate variables used in association analyses. Individual collection sites are shown in black. Grey plots above and right of the maps indicate average climate values by longitude and latitude across the study area, respectively.

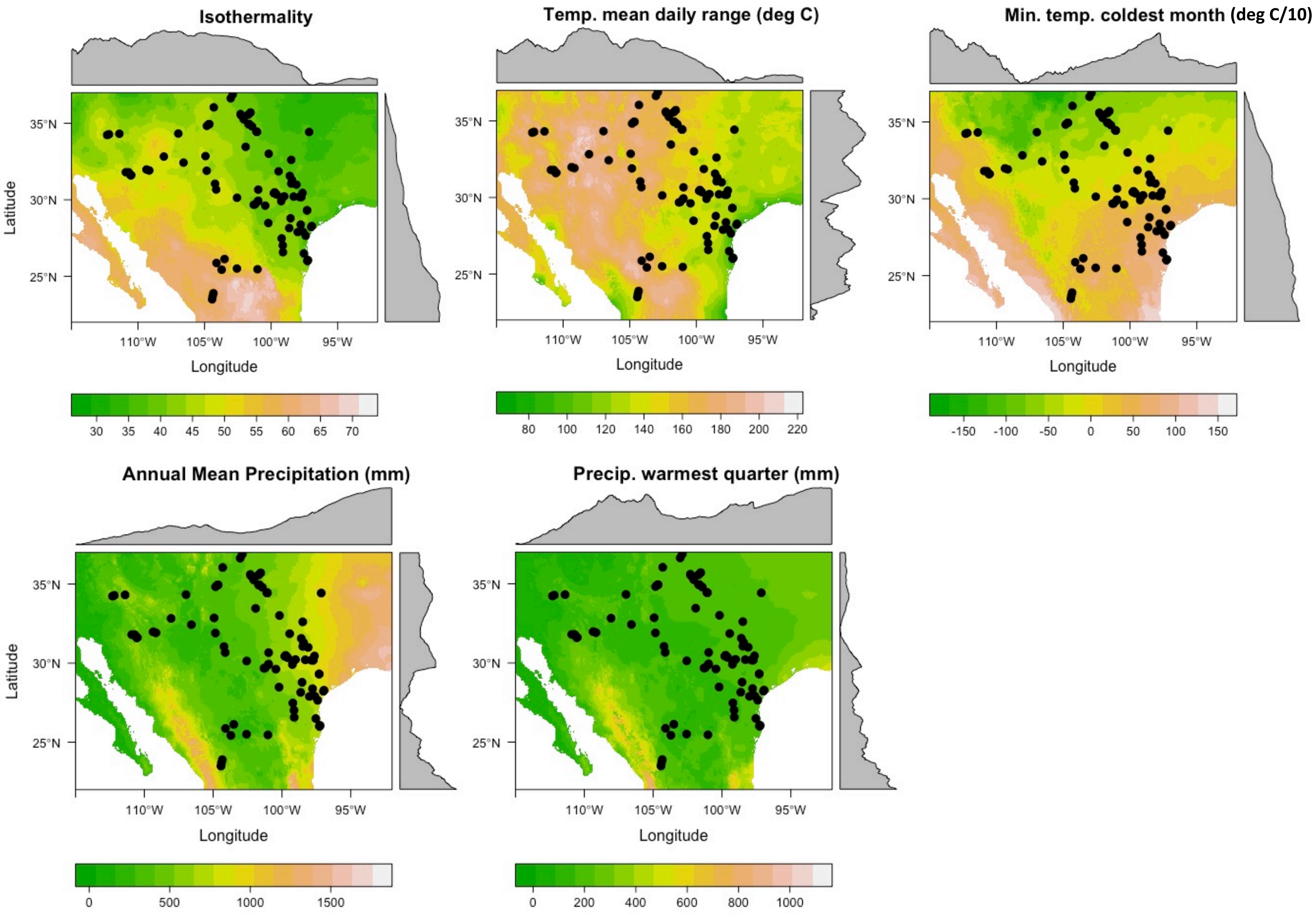

Fig. S6. Manhattan plots of SNP association with climate variables for *P.hallii* . Left: Q-Q plots show expected and observed p-value percentiles. Right: Association p-values for SNPs across the genome. Significantly associated snps are shown in red.

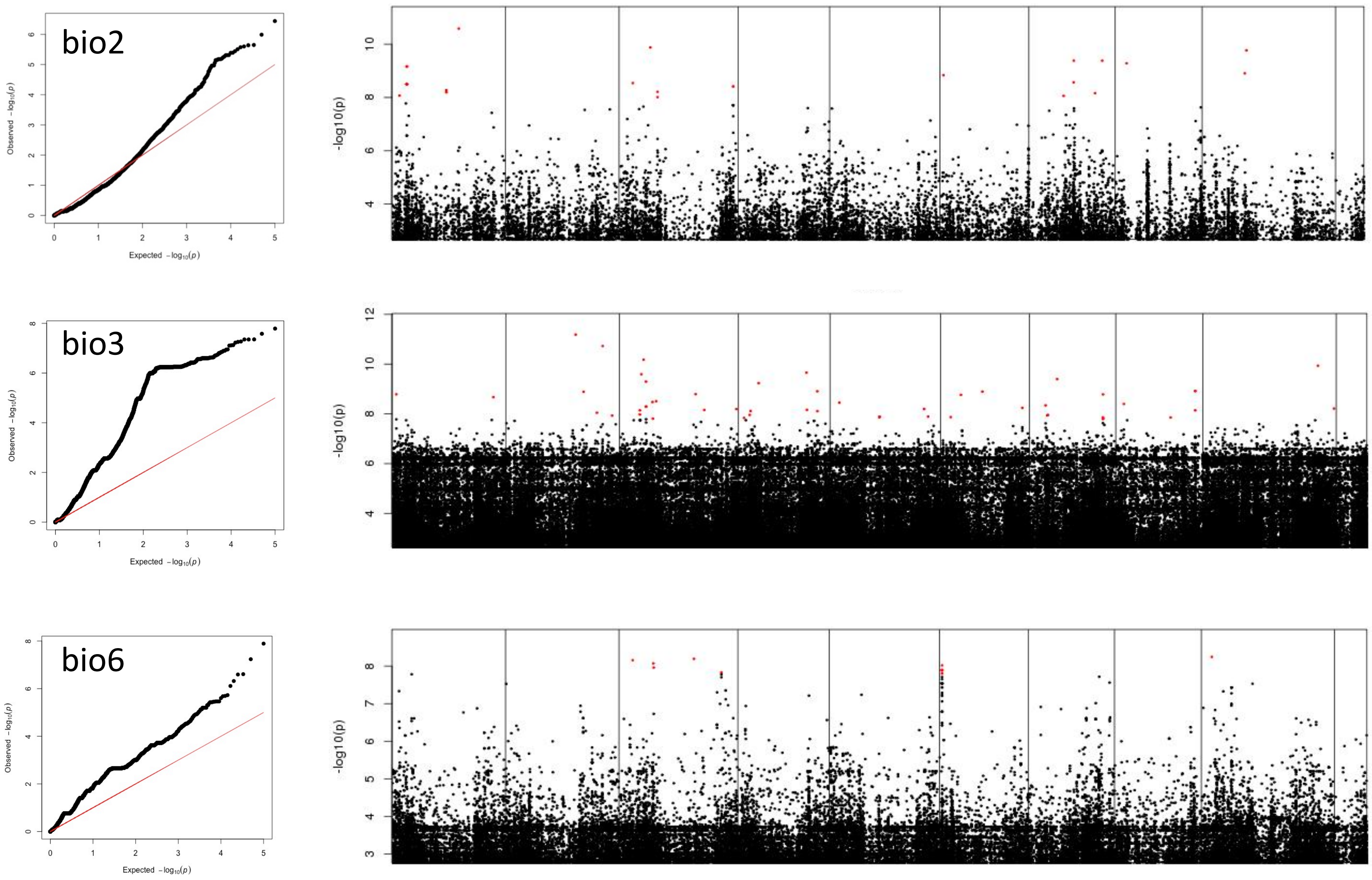

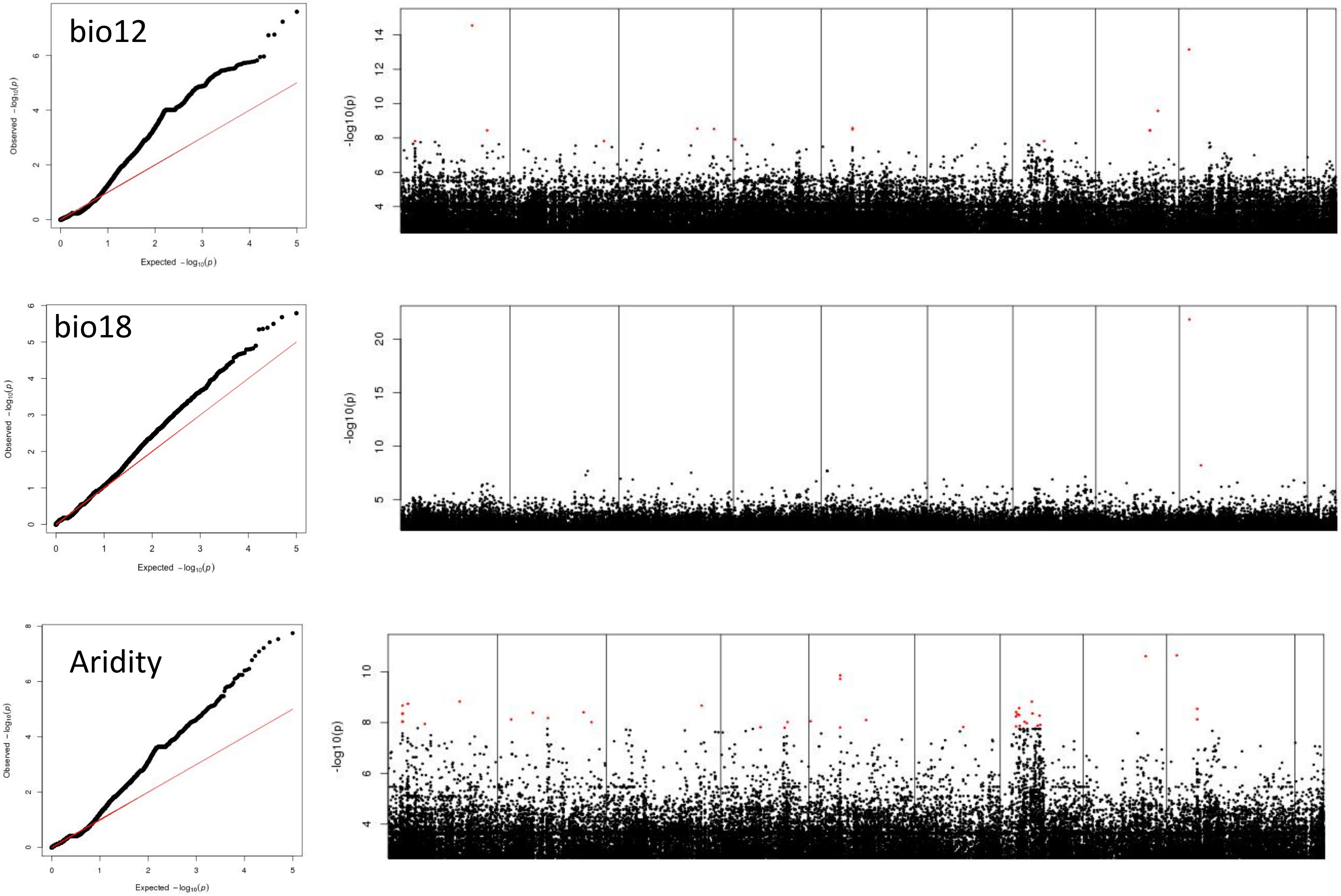

bio12
Observed −log10(p)
Expected −log10(p)
-log10(p)
bio18
Observed −log10(p)
Expected −log10(p)
-log10(p)
Aridity
Observed −log10(p)
Expected −log10(p)
-log10(p)

Fig. S6. Manhattan plots of SNP association with climate variables within *P.hallii var. hallii*. Left: Q-Q plots show expected and observed p-value percentiles. Right: Association p-values for SNPs across the genome. Significantly associated snps are shown in red.

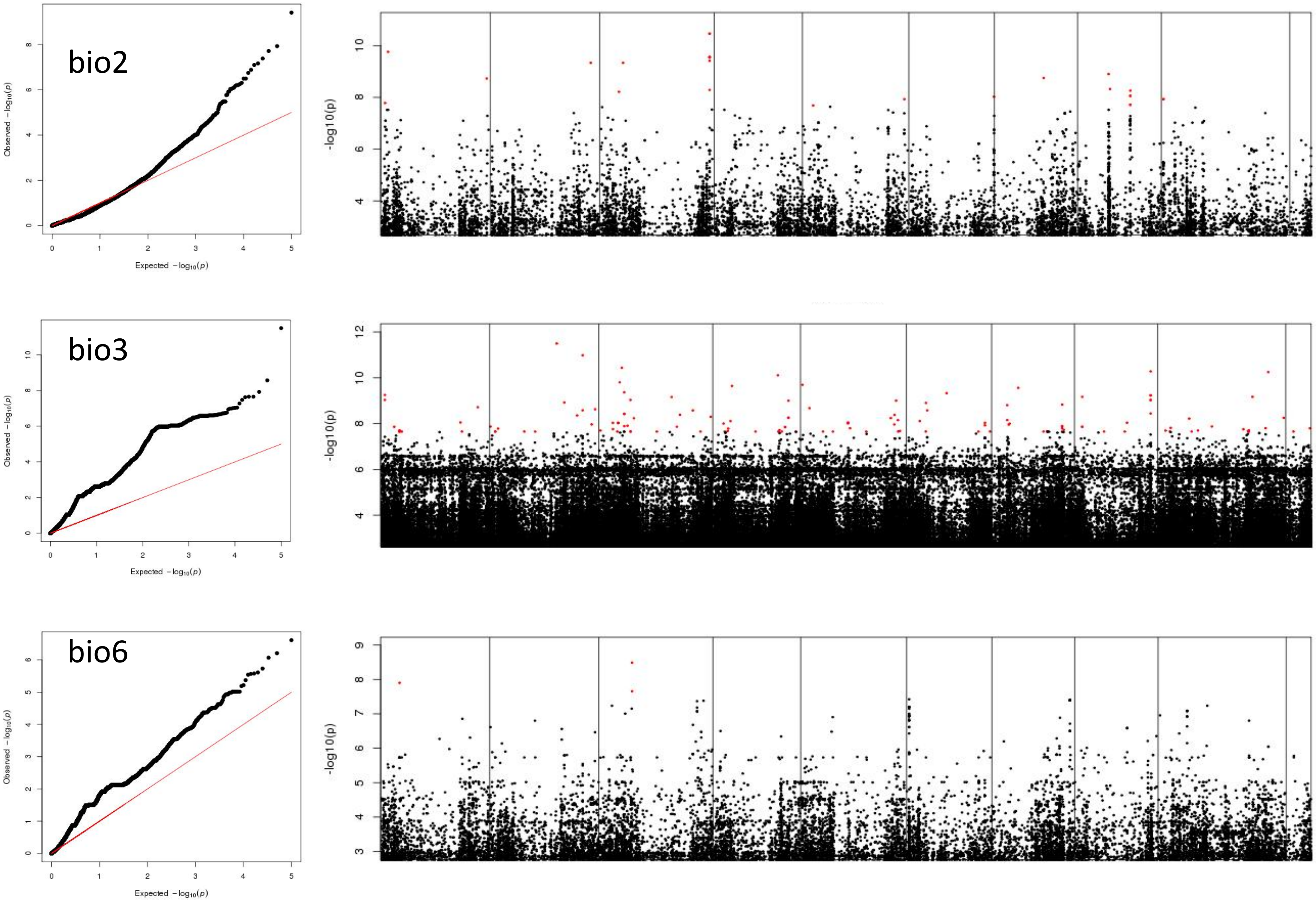

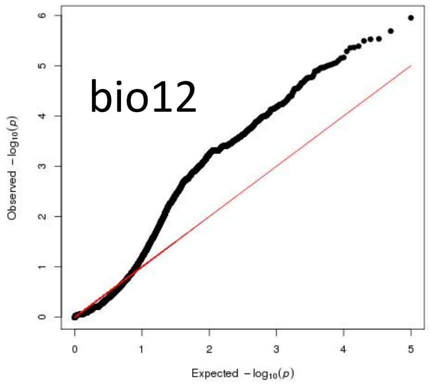
bio12
Observed −log10(p)
Expected −log10(p)

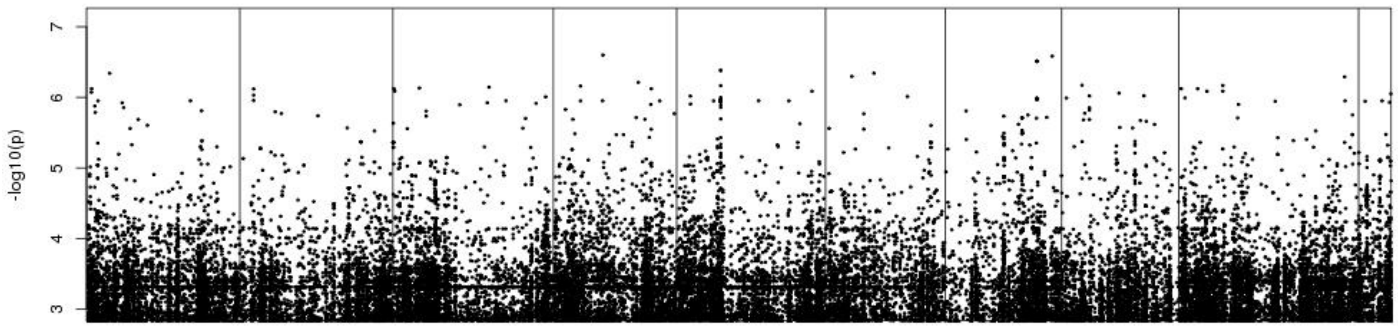
-log10(p)

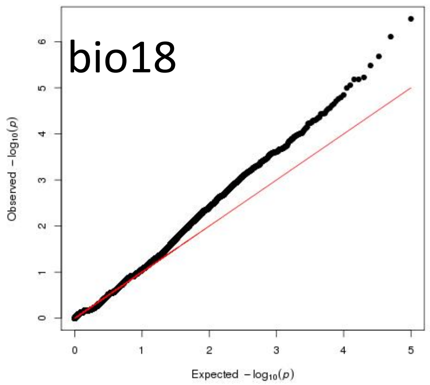
bio18
Observed −log10(p)
Expected −log10(p)

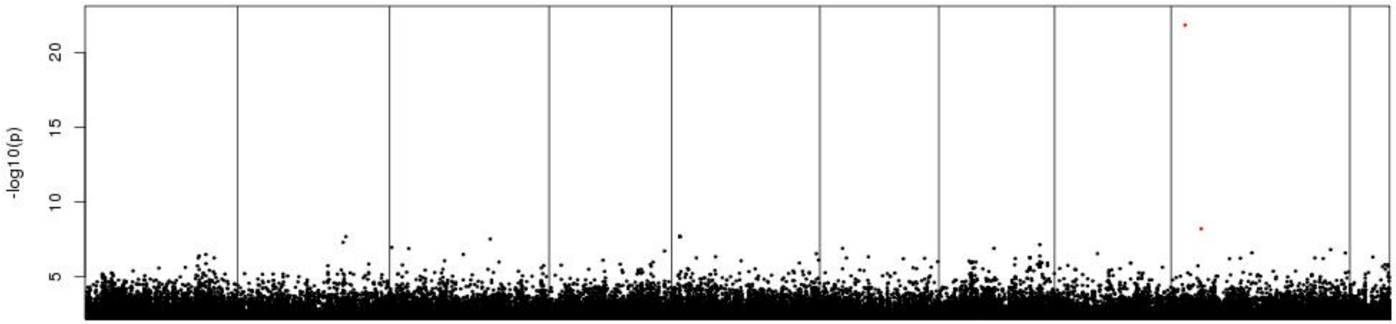
-log10(p)

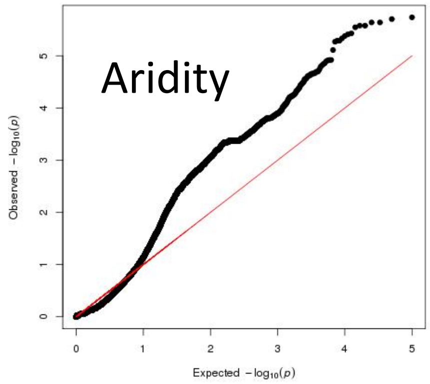
Aridity
Observed −log10(p)
Expected −log10(p)

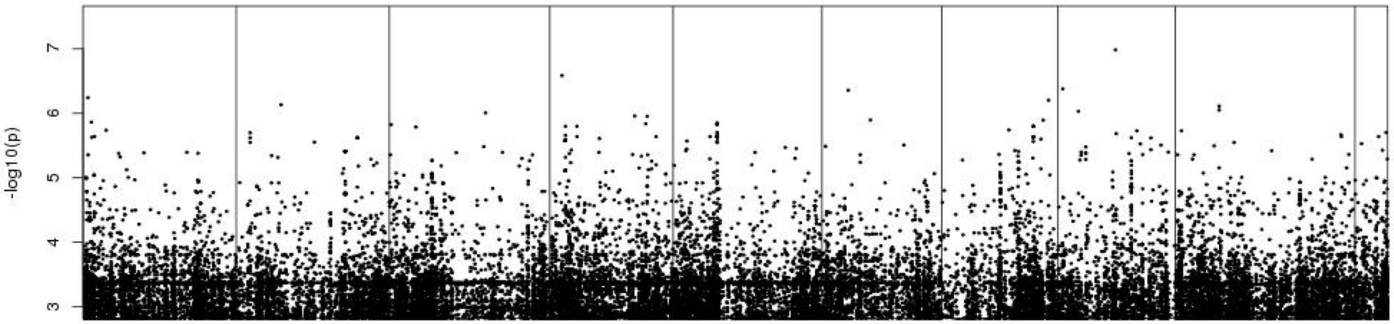
-log10(p)
